# Transvaginal evisceration after laparoscopic adrenalectomy in neurofibromatosis

**DOI:** 10.4103/0974-2700.62107

**Published:** 2010

**Authors:** Nereo Vettoretto, Luca Balestra, Lucio Taglietti, Maurizio Giovanetti

**Affiliations:** Department of General and Vascular Surgery, M. Mellini Hospital, Chiari (BS), Italy

**Keywords:** Complication, evisceration, laparoscopic adrenalectomy, surgery, vaginal

## Abstract

Transvaginal evisceration is a rare complication of hysterectomy. We describe this event following adrenalectomy for pheochromocytoma in a patient affected by neurofibromatosis. This is the first case reported in the literature following laparoscopic surgery. Prompt emergency intestinal reduction and vaginal cuff repair is required to prevent ischemia of the eviscerated bowel. Pneumoperitoneum, passage of stools, or an unknown connective tissue dysplasia due to genetic abnormalities might have contributed to this unpredictable event. The general surgeon must be aware of this rare but challenging gynecological complication.

## INTRODUCTION

Evisceration through the vaginal introitus is a rare emergency condition, which generally complicates hysterectomy. Since the first report in 1864 by Hyernaux, 113 cases have been described in the literature up to March 2009. Various causes have been ascribed to this dramatic event: obstetric maneuvers, vaginal instrumentation, rough coitus. Premenopausal cases are mostly due to precocious sexual intercourse after hysterectomy, whereas postmenopausal etiology is generally referred to a sudden increase in intra-abdominal pressure. Other causes may contribute to vaginal cuff weakness, among which are steroidal therapy and connective tissue diseases. We report the case of a postmenopausal women affected by Von- Recklinghausen syndrome and polymyositis which accused a vaginal evisceration shortly after a laparoscopic adrenalectomy for pheochromocytoma. To our knowledge, it is the first report of such a pathology occurring as a complication of laparoscopic surgery, and for this reason we judge it being worthy of reporting.

## CASE REPORT

A 64-year-old woman underwent right laparoscopic adrenalectomy for a 3 cm pheochromocytoma. Past medical history was remarkable for neurofibromatosis and polymyositis treated with 25 mg of prednisone daily; she underwent total transabdominal hysterectomy for fibromatosis 12 years before admittance. No complications were evidenced during laparoscopy, accomplished in the usual anti-Trendelembourg left flank decubitus with a peritoneal pressure of 13 mmHg. In third postoperative day, during the first passage of stools, she accused sharp hypogastric pain with the protrusion of 30 cm of ileal loops through the vagina. The surgeon on-call managed to replace manually the herniated intestine (which showed evidence of venous congestion) through a 5-cm defect in the vaginal cuff, and promptly indicated emergency laparotomy. Through a midline incision (over the previous hysterectomy scar), the bowel was checked for vitality and the vaginal cuff (showing signs of important seromucosal atrophy) repaired with single-layer absorbable interrupted suture after regularization of the impaired margins. Postoperative course was uneventful and the patient discharged on eighth postoperative day.

## DISCUSSION

The frequency of vaginal evisceration is very low. Among the 7286 hysterectomies collection by Hur, an incidence of 0.14% is reported (total and subtotal), with a peak rate of 4.93% after laparoscopic hysterectomy, pleading thermal cautery as a risk factor for the dehiscence of the cuff.[1] Another single institution case study (Iaco on 3593 hysterectomies) reports a rate of 0.28%, without the evidence of statistical difference between different routes of access (transabdominal, transvaginal, or laparoscopic) or the presence of a closed or unclosed cuff.[2] Ramirez, on a review of the literature on 59 eviscerations, highlights as risk factors a postmenopausal state, a transvaginal hysterectomy, and an increase in abdominal pressure.[3] Croak locates different sites of rupture between abdominal and vaginal hysterectomies, the former having lesions predominantly in the cuff, and the latter through a posterior wall enterocele,[4] as might happen after radical pelvic operations for cancer (i.e., cystectomy), in which rupture of the sac is the main etiology. Evisceration can occur even after subtotal hysterectomy, through the posterior fornix.[5]

All the authors agree on the need for emergency reduction and repair. The operation can be accomplished either in a transabdominal (open or laparoscopic) technique, by a transvaginal route, or by a combination of the two.[6] Most of the patients described in the literature had a late evisceration (median time 11 weeks) and were, therefore, admitted with an advanced vascular ischemia of the herniated bowel requiring resection.[7] Manual reduction in the abdomen can be accomplished only in the early stages of evisceration, like in our case in which the patient was hospitalized for surgery; loop necrosis can be avoided whenever the maneuver is made promptly and in a sterile fashion.

Although pelvic surgery (both oncological and functional) has been described as a risk factor for evisceration (cases are reported following pelvic floor repair or prolapse procedures, proctectomy, cystectomy),[8] we found no references regarding laparoscopic abdominal surger y as a cause of increased pressure and consequent vaginal rupture. In the present case, this event followed the surgical act by 3 days, and was triggered by the passage of stools. We cannot, therefore, strictly ascribe to pneumoperitoneum the primary etiology of the vaginal cuff 's rupture, even if the two events occurred too close to be independent from each other.

The vaginal atrophy, as a predisposing factor to cuff 's dehiscence, may be attributed to age, to a postmenopausal condition, but also to the systemic diseases affecting this patient. In fact, steroidal drugs have been advocated as a cause of weakness and atrophy of the mucosa,[9] and this subject had a long-term history of prednisone therapy, due to a polymyositis, rarely associated to Von Recklinghausen's disease. Moreover, connections between microdeletions of a certain type of NF1 gene might cause connective tissue dysplasia in neurofibromatosis’ patients;[10] unfortunately, we cannot ascertain the patient's genotype as she refused to be subjected to further genetic studies.

## CONCLUSION

We can acknowledge that multiple factors including pneumoperitoneum-induced increase in abdominal pressure, postoperative ileum, effort for the passage of stools, tissue weakness due to a combination of neurofibromatosis’ related connective tissue abnormality, and steroidal therapy might have competed in causing this infrequent but dangerous postoperative complication, of which the general surgeon must be aware.
